# Supplementary material for: Starchy Vegetables and Metabolic Syndrome in Costa Rica
Source: Nutrients. 2021 May 13;13(5):1639. doi: 10.3390/nu13051639 (PMC8152504; doi:10.3390/nu13051639)
Supplement: Supplementary file 1 [file nutrients-13-01639-s001.zip › nutrients-1213339-SI.pdf]

nutrients

MDPI

Supplementary Tables:

Supplementary Table S1. Characteristics of the study population without metabolic syndrome across quintiles of total starchy vegetables consumption (n = 1123).

Supplementary Table S1. Characteristics of the study population without metabolic syndrome across quintiles of total starchy vegetables consumption (n = 1123).

	Quintiles of starchy vegetables				
	1 (n = 224)	2 (n = 225)	3 (n = 225)	4 (n = 225)	5 (n = 224)
Total starchy vegetables[1]	24.40 ± 12.94	44.38 ± 8.79	59.76 ± 8.01	82.23 ± 14.10	128.90 ± 44.39
Age (year)[3]	53.97 ± 11.44	54.00 ± 11.09	54.48 ± 10.99	55.76 ± 11.29	60.08 ± 11.96
Women (%)	9.38	12.00	14.22	21.78	28.57
Urban residence (%)	39.73	41.33	43.11	37.33	44.64
Income ($/month)[2]	559.54 ± 439.27	579.08 ± 449.90	596.97 ± 403.42	635.97 ± 429.69	559.19 ± 455.42
BMI (kg/m^2)[1]	24.80 ± 3.35	24.96 ± 3.41	24.89 ± 3.51	24.66 ± 3.39	24.35 ± 3.39
Waist/hip ratio	0.95 ± 0.07	0.94 ± 0.07	0.94 ± 0.07	0.93 ± 0.07	0.92 ± 0.08
Current smoker (%)	38.39	32.89	20.44	21.33	17.86
Hypertension history (%)	11.61	13.78	12.89	17.78	12.95
High cholesterol history (%)[2]	18.30	24.11	16.00	20.00	23.21
Alcohol (gm)	10.52 ± 19.00	7.88 ± 17.73	6.79 ± 15.19	4.44 ± 8.97	4.40 ± 10.68
Systolic blood pressure (mm Hg)	127.86 ± 22.14	128.03 ± 18.29	128.76 ± 21.24	129.48 ± 20.95	132.95 ± 23.46
Diastolic blood pressure (mm Hg)	78.98 ± 9.69	79.43 ± 9.72	79.63 ± 10.67	78.88 ± 9.93	78.66 ± 10.19
HDL-C (mg/dL)[2]	42.24 ± 9.13	41.35 ± 8.08	41.83 ± 9.28	42.60 ± 9.07	43.80 ± 10.57
LDL-C (mg/dL)[2]	130.30 ± 37.43	131.03 ± 34.20	127.74 ± 33.08	132.08 ± 32.04	133.03 ± 44.08
Fasting blood glucose (mg/dL)[2]	74.09 ± 17.23	72.32 ± 12.30	72.31 ± 12.58	71.45 ± 12.12	71.72 ± 13.09
Total triglycerides (mg/dL)[2]	189.24 ± 106.54	202.20 ± 145.14	190.35 ± 117.43	201.29 ± 140.99	185.38 ± 120.84
EE on daily activity (METs/d)	38.92 ± 20.14	36.94 ± 16.59	37.31 ± 17.81	38.11 ± 19.58	34.35 ± 15.78
**Metabolic syndrome components (%)**					
Abdominal obesity (waist circumference ≥ 102 cm in men or 88 cm in women)	2.68	3.11	3.56	4.44	3.13
Hypertriglyceridemia (triglycerides ≥ 150 mg/dL)[2]	55.16	51.79	51.34	55.56	52.94

Low HDL cholesterol (high-density lipoprotein cholesterol < 40 mg/dL in men or 50 mg/dlL in women)[2]	45.50	45.95	51.36	45.50	46.12
High blood pressure (blood pressure ≥ 130 mm Hg systolic blood pressure or ≥ 85 mm Hg diastolic blood pressure)	41.07	45.33	46.22	46.67	51.34
High glucose (fasting glucose ≥ 100 mg/dL)[2]	2.69	0.89	0.89	0.44	1.34
**Dietary variables**					
Total energy intake (kcal/day)	2687.36 ± 846.59	2597.26 ± 765.04	2574.49 ± 628.53	2371.30 ± 588.59	2185.04 ± 588.24
Total fat (% of energy)	32.08 ± 7.04	32.07 ± 6.07	32.34 ± 6.06	31.55 ± 5.00	31.58 ± 5.69
Saturated fat (% of energy)	10.72 ± 2.95	10.54 ± 2.64	10.55 ± 2.70	10.29 ± 2.54	9.99 ± 2.69
Monounsaturated fat (% of energy)	12.22 ± 4.85	12.22 ± 4.21	12.32 ± 4.35	11.43 ± 2.91	11.62 ± 3.91
Polyunsaturated fat (% of energy)	5.77 ± 1.95	5.98 ± 1.79	6.06 ± 1.89	6.40 ± 1.88	6.40 ± 2.22
Trans fat (% of energy)	1.30 ± 0.67	1.24 ± 0.58	1.33 ± 0.66	1.33 ± 0.61	1.33 ± 0.66
Carbohydrate (% of energy)	54.17 ± 8.41	54.91 ± 7.42	54.89 ± 7.56	56.24 ± 6.35	56.10 ± 7.21
Protein (% of energy)	12.72 ± 2.28	12.60 ± 2.06	12.73 ± 2.00	12.78 ± 1.80	12.99 ± 2.12
Cholesterol (mg/kJ)	133.34 ± 74.61	119.34 ± 52.58	115.39 ± 41.84	116.32 ± 39.56	113.44 ± 48.01
Fiber (g/day)	21.60 ± 6.24	22.27 ± 5.99	22.95 ± 5.78	23.30 ± 5.17	24.43 ± 6.20
Total starchy vegetables (g/day)	22.79 ± 9.28	43.97 ± 5.24	60.40 ± 4.77	82.67 ± 8.09	140.60 ± 44.99
French fries	3.28 ± 16.26	8.62 ± 32.38	7.56 ± 22.70	16.22 ± 50.78	40.55 ± 89.69
Baked potatoes	5.57 ± 6.39	10.32 ± 8.60	12.14 ± 10.48	21.61 ± 16.02	41.75 ± 40.34
Potato chips	0.62 ± 1.40	1.40 ± 3.63	1.36 ± 3.60	2.47 ± 6.71	2.50 ± 7.10
Purple sweet potatoes	1.30 ± 2.93	3.02 ± 4.38	4.07 ± 5.27	6.06 ± 8.19	16.39 ± 25.53
Plantain	5.10 ± 6.14	12.91 ± 11.79	21.40 ± 15.88	30.04 ± 22.06	52.78 ± 55.56
Corn	1.03 ± 3.92	1.59 ± 3.43	1.71 ± 2.83	2.94 ± 8.00	4.01 ± 8.68
Cassava	5.49 ± 5.66	9.62 ± 7.59	13.49 ± 10.31	17.51 ± 11.54	35.09 ± 30.38

[1] Medians ± IQRs. [2] 68, 1, 18, 82, 2, 6, 6, 18, 2 missing observations, respectively. [3] Means ± SDs (all such values).

Supplementary Table S2. Sensitivity analysis: Prevalence ratios of metabolic syndrome according to quintiles of total starchy vegetables, unhealthy starchy vegetables, healthy starchy vegetables consumption after excluding subjects with history of hypertension (n = 1404).

	Quintiles					
	1 (n = 280)	2 (n = 281)	3 (n = 281)	4 (n = 281)	5 (n = 281)	P for trend
**Total starchy vegetables**						
Median intake (g/day)[1]	25.37 ± 12.65	44.89 ± 9.39	61.77 ± 8.11	84.50 ± 14.59	131.60 ± 41.35	
Crude model	1.0	0.92 (0.71, 1.18)	0.94 (0.73, 1.21)	0.97 (0.76, 1.25)	1.11 (0.88, 1.40)	0.2069
Adjusted model[2]	1.0	0.86 (0.68, 1.09)	0.87 (0.68, 1.11)	0.83 (0.65, 1.05)	0.82 (0.65, 1.03)	0.1594
Fully adjusted[3]	1.0	0.88 (0.69, 1.13)	0.88 (0.69, 1.13)	0.85 (0.67, 1.08)	0.85 (0.67, 1.08)	0.2568
**Unhealthy starchy vegetables**						
Median intake (g/day)[1]	2.86 ± 5.19	11.03 ± 3.80	19.93 ± 5.01	33.57 ± 9.84	68.47 ± 43.47	
Crude model	1.0	0.83 (0.64, 1.09)	1.110(0.87, 1.40)	1.03 (0.81, 1.32)	1.09 (0.85, 1.39)	0.2047
Adjusted model[2]	1.0	0.83 (0.64, 1.07)	1.02 (0.81, 1.29)	0.97 (0.77, 1.23)	0.88 (0.69, 1.12)	0.4687
Fully adjusted[3]	1.0	0.83 (0.64, 1.08)	1.04 (0.82, 1.32)	0.96 (0.76, 1.22)	0.90 (0.71, 1.15)	0.4622
**Healthy starchy vegetables**						
Median intake (g/day)[1]	11.62 ± 7.67	24.28 ± 5.64	38.26 ± 7.25	53.67 ± 9.64	87.38 ± 40.32	
Crude model	1.0	0.97 (0.76, 1.24)	0.87 (0.68, 1.13)	0.93 (0.72, 1.19)	1.11 (0.88, 1.40)	0.3029
Adjusted model[2]	1.0	0.93 (0.74, 1.17)	0.82 (0.64, 1.05)	0.82 (0.64, 1.04)	0.85 (0.67, 1.06)	0.1674
Fully adjusted[3]	1.0	0.94 (0.74, 1.19)	0.83 (0.65, 1.07)	0.82 (0.64, 1.04)	0.88 (0.69, 1.11)	0.2741

[1] Medians ± IQRs. [2] The model is adjusted for age, sex, current smoking status, urban residence and income. [3] The model is additionally adjusted for history of hypertension, alcohol, and total energy expenditure.

Supplementary Table S3. Sensitivity analysis: Least square means of metabolic syndrome according to quintiles of total starchy vegetables, unhealthy starchy vegetables, healthy starchy vegetables consumption after excluding subjects with history of hypertension (n = 1404).

		Quintiles					
		1 (n = 280)	2 (n = 281)	3 (n = 281)	4 (n = 281)	5 (n = 281)	P for trend
**Total starchy vegetables**							
Median intake (g/day)		25.37 ± 12.65	44.89 ± 9.39	61.77 ± 8.11	84.50 ± 14.59	131.60 ± 41.35	
Waist circumference (cm)	Crude model	90.29	89.89	89.85	88.44	87.62	<0.0001
	Fully adjusted	90.04	89.60	89.67	88.61	88.18	0.0068
Triglycerides (mg/dL)[1]	Crude model	208.34	217.32	210.55	215.39	202.79	0.4442

	Fully adjusted	203.55	214.21	211.04	216.97	208.63	0.7689
HDL cholesterol (mg/dL)[2]	Crude model	40.47	40.07	40.42	41.43	42.23	0.0016
	Fully adjusted	40.60	40.30	40.65	41.41	41.65	0.0530
Systolic blood pressure (mm Hg)	Crude model	128.13	127.27	129.52	129.78	133.82	<0.0001
	Fully adjusted	129.54	128.25	129.97	129.81	130.95	0.1544
Diastolic blood pressure (mm Hg)	Crude model	80.06	79.13	79.65	78.75	79.36	0.4089
	Fully adjusted	79.93	78.97	79.53	78.88	79.63	0.8706
Fasting blood glucose (mg/dL)[3]	Crude model	80.32	74.64	73.90	73.83	73.91	0.0031
	Fully adjusted	80.48	74.59	73.90	73.74	73.89	0.0033
**Unhealthy starchy vegetables**							
Median intake (g/day)		2.86 ± 5.19	11.03 ± 3.80	19.93 ± 5.01	33.57 ± 9.84	68.47 ± 43.47	
Waist circumference (cm)	Crude model	89.89	89.94	89.57	89.15	87.54	0.0005
	Fully adjusted	89.55	89.53	89.48	89.27	88.26	0.0529
Triglycerides (mg/dL)[1]	Crude model	206.73	214.51	223.95	210.98	198.21	0.1437
	Fully adjusted	205.74	211.92	221.91	212.33	202.48	0.4309
HDL cholesterol (mg/dL)[2]	Crude model	39.83	41.03	40.53	41.04	42.20	0.0024
	Fully adjusted	40.09	41.19	40.56	41.08	41.70	0.0531
Systolic blood pressure (mm Hg)	Crude model	128.39	128.22	130.73	130.43	130.75	0.0921
	Fully adjusted	128.45	129.48	131.01	130.52	129.06	0.9626
Diastolic blood pressure (mm Hg)	Crude model	79.70	79.04	79.91	78.97	79.32	0.6831
	Fully adjusted	79.47	78.74	79.94	79.05	79.75	0.5587
Fasting blood glucose (mg/dL)[3]	Crude model	76.89	76.27	75.91	72.16	75.34	0.2288

	Fully adjusted	76.76	76.17	75.81	72.14	75.71	0.3821
**Healthy starchy vegetables**							
Median intake (g/day)		11.62 ± 7.67	24.28 ± 5.64	38.26 ± 7.25	53.67 ± 9.64	87.38 ± 40.32	
Waist circumference (cm)	Crude model	89.22	90.11	90.04	89.18	87.53	0.0036
	Fully adjusted	89.06	89.64	90.09	89.21	88.11	0.0803
Triglycerides (mg/dL)[1]	Crude model	214.93	201.65	220.34	209.34	208.18	0.7633
	Fully adjusted	208.84	198.09	220.39	212.24	214.87	0.3208
HDL cholesterol (mg/dL)[2]	Crude model	40.97	40.55	39.90	40.52	42.55	0.0118
	Fully adjusted	40.93	41.03	40.06	40.50	42.08	0.1110
Systolic blood pressure (mm Hg)	Crude model	128.42	128.60	127.78	129.72	134.00	<0.0001
	Fully adjusted	130.10	128.80	128.82	129.01	131.80	0.1124
Diastolic blood pressure (mm Hg)	Crude model	79.92	79.50	79.10	78.98	79.45	0.5508
	Fully adjusted	79.88	79.29	79.15	78.95	79.67	0.9010
Fasting blood glucose (mg/dL)[3]	Crude model	76.70	78.00	74.17	74.50	73.21	0.0091
	Fully adjusted	76.96	77.66	74.26	74.46	73.23	0.0108

[1] 6 missing observations. [2] 19 missing observations. [3] 2 missing observations.

Supplementary Table S4. Sensitivity Analysis: Least square means of metabolic syndrome components (blood pressures and fasting blood glucose) according to quintiles of total starchy vegetables, unhealthy starchy vegetables, healthy vegetables consumptions in crude and fully adjusted models excluding people with HTN and DM medications. (n = 1506)

		Quintiles					
		1 (n = 301)	2 (n = 301)	3 (n = 302)	4 (n = 301)	5 (n = 301)	P for trend
**Total starchy vegetables**							
Median intake (g/day)		23.91 ± 12.16	43.62 ± 9.08	60.10 ± 8.17	81.93 ± 13.67	129.11 ± 42.23	
Systolic blood pressure (mm Hg)	Crude model	129.81	129.78	130.63	131.57	134.90	0.0003

	Fully adjusted	131.28	130.33	131.33	130.83	132.41	0.3171
Diastolic blood pressure (mm Hg)	Crude model	81.00	80.39	80.41	79.91	80.19	0.3146
	Fully adjusted	80.95	80.28	80.29	79.96	80.85	0.9715
Fasting blood glucose (mg/dL)[1]	Crude model	80.52	74.68	75.46	73.76	74.01	0.0021
	Fully adjusted	81.09	74.35	75.17	73.20	74.08	0.0030
**Unhealthy starchy vegetables**							
Median intake (g/day)		2.73 ± 4.99	10.55 ± 3.62	19.20 ± 4.94	31.98 ± 9.03	65.09 ± 41.95	
Systolic blood pressure (mm Hg)	Crude model	130.44	129.47	132.42	132.31	132.05	0.1707
	Fully adjusted	130.98	130.74	132.68	131.92	129.86	0.3797
Diastolic blood pressure (mm Hg)	Crude model	80.88	80.31	80.76	79.96	79.97	0.2569
	Fully adjusted	80.98	79.93	80.93	80.23	80.24	0.5442
Fasting blood glucose (mg/dL)[1]	Crude model	77.33	77.21	76.21	72.03	75.65	0.1547
	Fully adjusted	77.31	76.58	75.92	72.20	75.72	0.2702
**Healthy starchy vegetables**							
Median intake (g/day)		10.98 ± 7.56	23.86 ± 5.63	37.30 ± 7.35	52.16 ± 9.75	85.16 ± 36.49	
Systolic blood pressure (mm Hg)	Crude model	130.22	130.75	129.14	131.38	135.21	0.0006
	Fully adjusted	131.93	130.70	130.04	130.47	133.08	0.2904
Diastolic blood pressure (mm Hg)	Crude model	81.09	80.48	79.82	80.14	80.36	0.4572
	Fully adjusted	81.18	80.39	79.88	80.06	80.83	0.8746
Fasting blood glucose (mg/dL)[1]	Crude model	77.27	77.97	74.45	75.37	73.35	0.0095
	Fully adjusted	77.69	78.10	74.01	74.79	73.23	0.0066

[1] 3 missing observations.

Supplementary Table S5. Sensitivity analysis: Prevalence ratios of metabolic syndrome according to quintiles of total starchy vegetables, unhealthy starchy vegetables and healthy starchy vegetables consumption excluding people change in long term intake on fruits (n = 912).

	Quintiles					
	1 (n = 182)	2 (n = 183)	3 (n = 182)	4 (n = 183)	5 (n = 182)	P for trend
**Total starchy vegetables**						
Median intake (g/day)[1]	25.15 ± 13.00	45.06 ± 9.47	61.86 ± 8.98	84.66 ± 13.82	128.04 ± 39.57	
Crude model	1.0	1.11 (0.84, 1.47)	1.18 (0.90, 1.56)	1.28 (0.98, 1.67)	1.48 (1.15, 1.91)	0.0007
Adjusted model[2]	1.0	1.08 (0.83, 1.41)	1.17 (0.89, 1.52)	1.10 (0.85, 1.42)	1.18 (0.92, 1.51)	0.2332
Fully adjusted[3]	1.0	1.08 (0.83, 1.42)	1.11 (0.85, 1.44)	1.05 (0.81, 1.35)	1.18 (0.92, 1.51)	0.3866
**Unhealthy starchy vegetables**						
Median intake (g/day)[1]	2.31 ± 4.66	10.95 ± 3.71	20.17 ± 5.21	35.19 ± 10.23	70.36 ± 36.63	
Crude model	1.0	0.92 (0.69, 1.22)	1.25 (0.97, 1.62)	1.23 (0.95, 1.59)	1.28 (0.99, 1.65)	0.0158
Adjusted model[2]	1.0	0.87 (0.66, 1.14)	1.12 (0.88, 1.43)	1.12 (0.88, 1.43)	1.04 (0.81, 1.33)	0.4004
Fully adjusted[3]	1.0	0.91 (0.68, 1.20)	1.08 (0.84, 1.38)	1.06 (0.83, 1.36)	1.01 (0.79, 1.30)	0.6724
**Healthy starchy vegetables**						
Median intake (g/day)[1]	11.78 ± 7.11	24.64 ± 6.00	38.05 ± 7.23	54.17 ± 9.96	84.99 ± 32.29	
Crude model	1.0	0.97 (0.74, 1.26)	0.90 (0.68, 1.18)	1.10 (0.85, 1.41)	1.30 (1.03, 1.65)	0.0040
Adjusted model[2]	1.0	0.94 (0.74, 1.20)	0.88 (0.67, 1.13)	0.94 (0.74, 1.19)	1.03 (0.83, 1.30)	0.4743
Fully adjusted[3]	1.0	0.98 (0.78, 1.23)	0.90 (0.72, 1.13)	0.93 (0.76, 1.15)	1.04 (0.85, 1.27)	0.6189

[1] Medians ± IQRs. [2] The model is adjusted for age, sex, current smoking status, urban residence and income. [3] The model is additionally adjusted for history of hypertension, alcohol, and total energy expenditure.

Supplementary Table S6. Sensitivity analysis: Least square means of metabolic syndrome according to quintiles of total starchy vegetables, unhealthy starchy vegetables and healthy starchy vegetables consumption excluding people change in long term intake on fruits (n = 912).

		Quintiles					
		1 (n = 182)	2 (n = 183)	3 (n = 182)	4 (n = 183)	5 (n = 182)	P for trend
**Total starchy vegetables**							
Median intake (g/day)		25.15 ± 13.00	45.06 ± 9.47	61.86 ± 8.98	84.66 ± 13.82	128.04 ± 39.57	
Adjusted waist circumference (cm)[1]	Crude model	102.76	102.38	102.25	101.51	100.42	<0.0001
	Fully adjusted	102.08	101.76	102.10	102.02	101.36	0.1471
Triglycerides (mg/dL)[2]	Crude model	208.00	208.92	215.35	222.50	212.24	0.5800

	Fully adjusted	204.07	205.99	215.89	224.40	216.66	0.2140
HDL cholesterol (mg/dL)[2]	Crude model	40.65	40.02	40.09	41.31	41.98	0.0304
	Fully adjusted	40.62	40.27	40.32	41.30	41.55	0.1332
Systolic blood pressure (mm/Hg)	Crude model	134.30	132.84	134.40	136.72	139.32	0.0062
	Fully adjusted	136.38	134.43	134.89	135.34	136.55	0.6540
Diastolic blood pressure (mm/Hg)	Crude model	81.65	80.94	81.95	81.67	82.39	0.3688
	Fully adjusted	81.85	81.10	81.83	81.41	82.42	0.4646
Fasting blood glucose (mg/dL)[2]	Crude model	76.20	74.49	77.90	74.45	73.74	0.2010
	Fully adjusted	76.22	74.60	77.96	74.24	73.75	0.1960
**Unhealthy starchy vegetables**							
Median intake (g/day)		2.31 ± 4.66	10.95 ± 3.71	20.17 ± 5.21	35.19 ± 10.23	70.36 ± 36.63	
Adjusted waist circumference (cm)[1]	Crude model	102.42	102.51	101.82	101.98	100.59	0.0003
	Fully adjusted	101.84	102.19	101.89	101.90	101.51	0.2377
Triglycerides (mg/dL)[2]	Crude model	207.12	213.66	233.13	205.26	207.70	0.5452
	Fully adjusted	207.93	211.72	231.82	204.28	211.14	0.7434
HDL cholesterol (mg/dL)[2]	Crude model	40.10	40.86	41.09	40.28	41.73	0.1377
	Fully adjusted	40.36	40.81	41.03	40.36	41.52	0.2881
Systolic blood pressure (mm/Hg)	Crude model	133.32	133.07	137.59	137.27	136.33	0.1569
	Fully adjusted	134.53	135.35	137.40	136.04	134.25	0.6129
Diastolic blood pressure (mm/Hg)	Crude model	81.80	80.66	82.16	82.31	81.68	0.7311
	Fully adjusted	81.93	81.06	82.25	81.79	81.58	0.9148

Fasting blood glucose (mg/dL)[2]	Crude model	75.61	76.20	76.13	74.48	74.36	0.3207
	Fully adjusted	75.82	76.32	75.90	74.23	74.50	0.3179
**Healthy starchy vegetables**							
Median intake (g/day)		11.78 ± 7.11	24.64 ± 6.00	38.05 ± 7.23	54.17 ± 9.96	84.99 ± 32.29	
Adjusted waist circumference (cm)[1]	Crude model	102.92	102.32	102.01	101.33	100.75	<0.0001
	Fully adjusted	102.02	101.67	102.11	101.87	101.66	0.5415
Triglycerides (mg/dL)[2]	Crude model	212.91	205.66	213.61	212.52	222.29	0.3210
	Fully adjusted	204.80	203.57	214.93	216.55	227.18	0.0491
HDL cholesterol (mg/dL)[2]	Crude model	40.56	40.24	40.29	40.25	42.71	0.0084
	Fully adjusted	40.57	40.48	40.43	40.29	42.28	0.0477
Systolic blood pressure (mm/Hg)	Crude model	135.58	133.13	133.69	136.71	138.47	0.0515
	Fully adjusted	137.68	133.98	134.77	134.87	136.29	0.9137
Diastolic blood pressure (mm/Hg)	Crude model	82.42	80.79	81.13	82.69	81.57	0.9550
	Fully adjusted	82.23	80.80	81.33	82.34	81.91	0.6875
Fasting blood glucose (mg/dL)[2]	Crude model	77.04	75.52	74.87	76.55	72.79	0.0689
	Fully adjusted	76.98	75.39	75.14	76.62	72.64	0.0724

[1] Waist circumference is adjusted for age and BMI. [2] 4, 14, 1 missing observations, respectively.

Supplementary Table S7. Sensitivity analysis: Prevalence ratios of metabolic syndrome according to quintiles of total starchy vegetables, unhealthy starchy vegetables and healthy starchy vegetables consumption excluding people change in long term intake on vegetables (n = 1005).

	Quintiles					
	1 (n = 201)	2 (n = 201)	3 (n = 201)	4 (n = 201)	5 (n = 201)	P for trend
**Total starchy vegetables**						
Median intake (g/day)[1]	24.70 ± 34.97	45.31 ± 19.49	62.05 ± 17.13	84.69 ± 28.95	129.87 ± 260.86	
Crude model	1.0	1.00 (0.77, 1.30)	1.07 (0.83, 1.39)	1.14 (0.89, 1.47)	1.42 (1.13, 1.79)	0.0004

Adjusted model[2]	1.0	0.94 (0.73, 1.20)	1.04 (0.82, 1.34)	0.97 (0.76, 1.24)	1.05 (0.83, 1.32)	0.4787
Fully adjusted[3]	1.0	0.97 (0.75, 1.25)	1.00 (0.79, 1.28)	0.96 (0.75, 1.22)	1.13 (0.90, 1.42)	0.2239
**Unhealthy starchy vegetables**						
Median intake (g/day)[1]	2.96 ± 4.98	10.71 ± 3.42	19.23 ± 5.43	32.76 ± 9.02	68.54 ± 36.80	
Crude model	1.0	0.93 (0.71, 1.22)	1.17 (0.91, 1.49)	1.17 (0.91, 1.49)	1.29 (1.02, 1.64)	0.0075
Adjusted model[2]	1.0	0.88 (0.68, 1.13)	1.09 (0.86, 1.38)	1.07 (0.84, 1.35)	1.05 (0.84, 1.33)	0.3291
Fully adjusted[3]	1.0	0.93 (0.72, 1.21)	1.07 (0.84, 1.35)	1.01 (0.80, 1.27)	1.05 (0.83, 1.32)	0.2533
**Healthy starchy vegetables**						
Median intake (g/day)[1]	11.90 ± 6.38	24.64 ± 5.42	38.29 ± 7.13	55.27 ± 9.42	85.44 ± 35.40	
Crude model	1.0	1.01 (0.79, 1.31)	0.96 (0.74, 1.24)	1.14 (0.89, 1.45)	1.30 (1.03, 1.64)	0.0066
Adjusted model[2]	1.0	0.97 (0.76, 1.24)	0.88 (0.69, 1.13)	1.00 (0.78, 1.26)	1.02 (0.81, 1.29)	0.8761
Fully adjusted[3]	1.0	0.97 (0.76, 1.23)	0.89 (0.69, 1.14)	0.98 (0.78, 1.24)	1.05 (0.84, 1.32)	0.9727

[1] Medians ± IQRs. [2] The model is adjusted for age, sex, current smoking status, urban residence and income. [3] The model is additionally adjusted for history of hypertension, alcohol, and total energy expenditure.

Supplementary Table S8. Sensitivity analysis: Least square means of metabolic syndrome according to quintiles of total starchy vegetables, unhealthy starchy vegetables and healthy starchy vegetables consumption excluding people change in long term intake on vegetables (n = 1005).

		Quintiles					
		1 (n = 201)	2 (n = 201)	3 (n = 201)	4 (n = 201)	5 (n = 201)	P for trend
**Total starchy vegetables**							
Median intake (g/day)		24.70 ± 34.97	45.31 ± 19.49	62.05 ± 17.13	84.69 ± 28.95	129.87 ± 260.86	
Adjusted waist circumference (cm)[1]	Crude model	105.14	104.36	104.58	104.02	102.52	<0.0001
	Fully adjusted	104.38	104.04	104.32	104.31	103.56	0.0881
Triglycerides (mg/dL)[2]	Crude model	218.88	200.59	213.96	211.33	213.18	0.9844
	Fully adjusted	215.29	200.63	214.55	211.50	215.97	0.6189
HDL cholesterol (mg/dL)[2]	Crude model	40.52	40.54	40.49	41.10	42.52	0.0084
	Fully adjusted	40.76	40.80	40.88	41.09	41.64	0.2553
Systolic blood pressure (mm/Hg)	Crude model	134.02	132.42	133.05	136.58	140.66	0.0001

	Fully adjusted	136.12	133.92	133.68	135.33	137.69	0.1660
Diastolic blood pressure (mm/Hg)	Crude model	82.16	80.70	81.46	82.06	82.13	0.5511
	Fully adjusted	82.18	81.00	81.18	81.81	82.34	0.5123
Fasting blood glucose (mg/dL)[2]	Crude model	77.86	73.61	77.86	75.47	73.94	0.1321
	Fully adjusted	77.75	73.80	77.85	75.25	74.09	0.1616
**Unhealthy starchy vegetables**							
Median intake (g/day)		2.96 ± 4.98	10.71 ± 3.42	19.23 ± 5.43	32.76 ± 9.02	68.54 ± 36.80	
Adjusted waist circumference (cm)[1]	Crude model	104.77	104.80	104.28	104.22	102.54	<0.0001
	Fully adjusted	104.24	104.44	104.19	104.14	103.60	0.0533
Triglycerides (mg/dL)[2]	Crude model	216.27	210.52	222.77	209.13	199.21	0.0954
	Fully adjusted	217.90	210.14	220.71	208.19	200.97	0.1243
HDL cholesterol (mg/dL)[2]	Crude model	39.90	41.18	41.11	40.92	42.07	0.0344
	Fully adjusted	40.20	41.50	41.06	40.89	41.52	0.3145
Systolic blood pressure (mm/Hg)	Crude model	133.91	133.59	136.00	136.93	136.33	0.1805
	Fully adjusted	134.84	136.04	136.35	135.02	134.49	0.5165
Diastolic blood pressure (mm/Hg)	Crude model	82.12	81.42	81.89	81.28	81.82	0.9081
	Fully adjusted	82.07	81.70	82.11	80.73	81.91	0.8058
Fasting blood glucose (mg/dL)[2]	Crude model	74.46	77.90	75.99	74.93	75.46	0.7539
	Fully adjusted	74.60	78.00	75.80	74.65	75.69	0.8008
**Healthy starchy vegetables**							
Median intake (g/day)		11.90 ± 6.38	24.64 ± 5.42	38.29 ± 7.13	55.27 ± 9.42	85.44 ± 35.40	
Adjusted waist circumference (cm)[1]	Crude model	104.90	104.96	103.89	104.11	102.75	<0.0001
	Fully adjusted	104.13	104.24	104.04	104.34	103.85	0.5384

Triglycerides (mg/dL)[2]	Crude model	216.70	202.43	208.01	211.66	219.11	0.4646
	Fully adjusted	210.38	200.89	209.61	213.85	223.20	0.1152
HDL cholesterol (mg/dL)[2]	Crude model	40.75	40.76	40.04	40.36	43.23	0.0031
	Fully adjusted	40.86	41.26	40.16	40.46	42.42	0.1024
Systolic blood pressure (mm/Hg)	Crude model	134.38	132.73	134.19	136.54	138.90	0.0053
	Fully adjusted	136.96	132.99	135.09	135.11	136.59	0.5602
Diastolic blood pressure (mm/Hg)	Crude model	82.36	80.78	81.21	82.76	81.41	0.9916
	Fully adjusted	82.29	80.58	81.39	82.42	81.83	0.6612
Fasting blood glucose (mg/dL)[2]	Crude model	77.15	77.22	74.13	76.12	74.12	0.1144
	Fully adjusted	76.93	77.06	74.32	76.07	74.35	0.1965

[1] Waist circumference is adjusted for age and BMI. [2] 3, 9, 1 missing observations, respectively.
